# Immune-checkpoint protein VISTA critically regulates the IL-23/IL-17 inflammatory axis

**DOI:** 10.1038/s41598-017-01411-1

**Published:** 2017-05-03

**Authors:** Na Li, Wenwen Xu, Ying Yuan, Natarajan Ayithan, Yasutomo Imai, Xuesong Wu, Halli Miller, Michael Olson, Yunfeng Feng, Yina H. Huang, Mary Jo Turk, Samuel T. Hwang, Subramaniam Malarkannan, Li Wang

**Affiliations:** 1 Department of Microbiology and Immunology, Milwaukee, WI 53226 USA; 2 Department of Dermatology, Medical College of Wisconsin, Milwaukee, WI 53226 USA; 3 Department of Medicine, Medical College of Wisconsin, Milwaukee, WI 53226 USA; 4 Department of Pediatrics, Medical College of Wisconsin, Milwaukee, WI 53226 USA; 5 Department of Blood Research Institute, Milwaukee, WI 53226 USA; 6 Department of Microbiology and Immunology, Geisel School of Medicine at Dartmouth, Hanover, New Hampshire USA; 7 Present Address: Shanghai University of Traditional Chinese Medicine, College of Pharmacy, Shanghai, 201203 P. R. China; 8 Present Address: Institute of Human Virology, University of Maryland School of Medicine, Baltimore, MD 21201 USA; 9 Present Address: Department of Dermatology, Hyogo College of Medicine 1-1, Mukogawa-cho, Nishinomiya, Hyogo 663-8501 Japan; 10 Present Address: Department of Dermatology, University of California Davis, Sacramento, CA 95816 USA; 11 Department of Histology and Embryology, Harbin Medical University, Harbin, 150086 P. R. China

## Abstract

V-domain Immunoglobulin Suppressor of T cell Activation (VISTA) is an inhibitory immune-checkpoint molecule that suppresses CD4^+^ and CD8^+^ T cell activation when expressed on antigen-presenting cells. *Vsir*^−/−^ mice developed loss of peripheral tolerance and multi-organ chronic inflammatory phenotypes. *Vsir*^−/−^ CD4^+^ and CD8^+^ T cells were hyper-responsive towards self- and foreign antigens. Whether or not VISTA regulates innate immunity is unknown. Using a murine model of psoriasis induced by TLR7 agonist imiquimod (IMQ), we show that VISTA deficiency exacerbated psoriasiform inflammation. Enhanced TLR7 signaling in *Vsir*^−/−^ dendritic cells (DCs) led to the hyper-activation of Erk1/2 and Jnk1/2, and augmented the production of IL-23. IL-23, in turn, promoted the expression of IL-17A in both TCRγδ^+^ T cells and CD4^+^ Th17 cells. Furthermore, VISTA regulates the peripheral homeostasis of CD27^−^ γδ T cells and their activation upon TCR-mediated or cytokine-mediated stimulation. IL-17A-producing CD27^−^ γδ T cells were expanded in the *Vsir*^−/−^ mice and amplified the inflammatory cascade. In conclusion, this study has demonstrated that VISTA critically regulates the inflammatory responses mediated by DCs and IL-17-producing TCRγδ^+^ and CD4^+^ Th17 T cells following TLR7 stimulation. Our finding provides a rationale for therapeutically enhancing VISTA-mediated pathways to benefit the treatment of autoimmune and inflammatory disorders.

## Introduction

V-domain Immunoglobulin Suppressor of T cell Activation (VISTA, gene name *Vsir*) is an inhibitory B7 family immune-checkpoint molecule^1^. Together with other T cell co-inhibitory receptors such as CTLA-4, PD-1, TIM3, and LAG3, these immune-checkpoint proteins play critical roles in maintaining peripheral tolerance and controlling immune responses against self and infectious agents, or cancer^2,3^.

The human and murine VISTA proteins share 90% identity and display similar expression patterns^4^. VISTA is constitutively expressed on CD11b^+^ myeloid dendritic cells (DCs), naïve CD4^+^ and CD8^+^ T cells, and Foxp3^+^CD4^+^ regulatory T cells. Similar to CTLA-4 and PD-1, VISTA controls peripheral tolerance and anti-tumor immunity^1,3,5^. VISTA expressed on APCs acts as a ligand to suppress the proliferation and cytokine production of both CD4^+^ and CD8^+^ T cells. VISTA expressed on CD4^+^ T cells also suppresses T cell activation in a T-cell autonomous manner^6^. *Vsir* knockout mice (*Vsir*^−/−^) developed loss of peripheral tolerance, manifested as spontaneous T cell activation, production of inflammatory cytokines and chemokines, and chronic multi-organ inflammation^7^. When bred onto an autoimmune-prone background, VISTA deficiency accelerated disease development in the experimental autoimmune encephalomyelitis (EAE) model^8^. Treatment with VISTA-blocking monoclonal antibody (mAb) enhanced T responses and anti-tumor immunity^1,8^. Our recent study has further demonstrated that VISTA and another B7 family immune-checkpoint PD-1 play non-redundant roles in controlling T cell responses^9^. Mice deficient for both genes developed the most severe inflammatory phenotypes, accompanied by spontaneous activation of CD4^+^ and CD8^+^ T cells. Combinational blockade of both VISTA and PD-1 proteins using blocking mAb led to synergized anti-tumor immune responses in murine models.

Irrespective of these findings, whether VISTA regulates innate immune responses is not known. To address this question, we have employed the imiquimod (IMQ)-induced murine model of psoriasis, where topical application of IMQ stimulates a network of innate immune cells, such as DCs and IL-17-producing γδ TCR^+^ T cells, leading to psoriasiform skin inflammation^10^. This murine model bears strong relevance to human psoriasis, which is also mediated by the IL-23/IL-17 inflammatory axis. Multiple studies have reported the presence of both IL-17-producing TCRγδ^+^ T cells (γδ T cells) and CD4^+^ Th17 cells in human psoriatic skin^11–15^. Treatment in human cancer patients with IMQ (Aldara^®^) has resulted in similar psoriasiform dermatitis, manifested as epidermal acanthosis and parakeratosis^16–18^.

In this study, using the IMQ-induced psoriasis model, we have demonstrated that VISTA plays a key role in suppressing the IL-23/IL-17-mediated inflammatory axis. VISTA inhibits the activation of DCs and the production of IL-23 following TLR7 stimulation. VISTA also regulates the activation of IL-17-producing γδ T cells and CD4^+^ Th17 T cells, as well as the peripheral homeostasis of CD27^−^ γδ T cell subsets that are pre-committed to produce IL-17A. Consequently, VISTA deficiency exacerbated psoriasiform inflammation. Taken together its role in suppressing CD4^+^ and CD8^+^ T cell activation, our study indicates that VISTA is a unique immune-checkpoint that regulates both innate and adaptive immune responses.

## Results

### *Vsir*^−/−^ mice developed exacerbated psoriasiform inflammation

**Figure 1:**
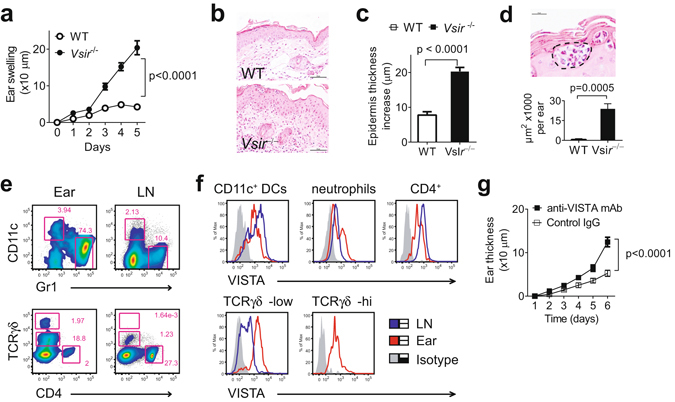
VISTA deficiency exacerbated the IMQ-induced psoriasiform inflammation. (**a**) WT and *Vsir*^−/−^ mice were topically treated daily on each ear with 3.5% IMQ cream for 5 days. Ear thickness was measured daily. Ear swelling is shown as the increase of ear thickness when compared to day 0, and expressed as mean ± SEM (n = 18). On day 5, ears were harvested and processed for H&E staining. A representative image is shown (**b**). Scale bars: 50 μm. (**c**) The epidermal thickness was measured by examining at least 20 random fields throughout the cross section of ear tissues. The increase of epidermal thickness was calculated by subtracting the average value of naive ears. Data are pooled from 3 ears and shown as mean ± SEM. (**d**) A representative image of Munro’s abscess in epidermis is shown (within the dashed lines). Scale bar: 50 μm. Areas of Munro’s abscess were measured from the entire cross section of ear tissues. Data are pooled from 3 ears and shown as ± SEM. (**e**) The number of CD11c^+^ DC, Gr1^+^ neutrophils, γδ TCR^+^ and CD4^+^ T cells in IMQ-treated ears and ear-draining LN in WT mice was examined by flow cytometry. (**f**) Surface VISTA expression on these cell types was examined by flow cytometry. Representative data from at least three independent experiments are shown. (**g**) WT mice were treated with VISTA-specific mAb or control Ig (250 μg, on day 0, 2, and 4), in addition to the 3.5% IMQ cream. Ear swelling is shown as mean ± SEM (n = 10).

To address the role of VISTA in regulating innate immunity, we examined IMQ-induced psoriasiform dermatitis in wild type (WT) and *Vsir*^−/−^ mice that were topically treated with 3.5% IMQ on both ears. Skin inflammatory response was quantified by measuring ear thickness. Our previous study reported chronic inflammatory phenotypes in aged (>10 month of age) *Vsir*^−/−^ mice^7^. We first examined untreated naïve *Vsir*^−/−^ mice (7–8 weeks of age) but did not observe any spontaneous skin inflammation (unpublished data). IMQ treatment in the *Vsir*^−/−^ mice resulted in more severe ear swelling when compared to WT mice (Fig. 1a). Histological analyses confirmed the development of severe epidermal acanthosis in the *Vsir*^−/−^ ear skin (Fig. 1b) and increased epidermal thickness (Fig. 1c). Furthermore, *Vsir*^−/−^ ears showed ~20 fold increase in the area of neutrophilic abscesses (Munro’s abscess), which is a histological hallmark in human psoriasis^18^ (Fig. 1d).

In addition to Gr1^+^ neutrophils, further examinations show that multiple other cell types were present in the WT psoriatic skin lesions, including CD11c^+^ DCs, CD4^+^ T cells, and γδ T cells (Fig. 1e). Similar lymphocyte populations were present in the ears of the *Vsir*^−/−^ mice (data not shown). VISTA was highly expressed on these cells types (Fig. 1f), all of which are known to regulate the development of IMQ-induced psoriasiform inflammation^17–20^. Both γδ^low^ and γδ^high^ T cells were present in the ear skin, whereas only γδ^low^ T cells were present in the ear-draining lymph nodes (LN). VISTA expression on γδ^low^ T cells was higher from inflamed ear skin than cells from the draining LN (Fig. 1f). To further determine whether the exacerbated psoriasiform inflammation was due to the pre-existing inflammatory environment in the *Vsir*^−/−^ mice, we treated mice with a VISTA-specific mAb^8^. Consistent with results from the *Vsir*^−/−^ mice, VISTA-specific mAb treatment significantly enhanced IMQ-induced psoriasiform inflammation (Fig. 1g), although the magnitude of disease was not as severe as those seen in the *Vsir*^−/−^ mice.

**Figure 2:**
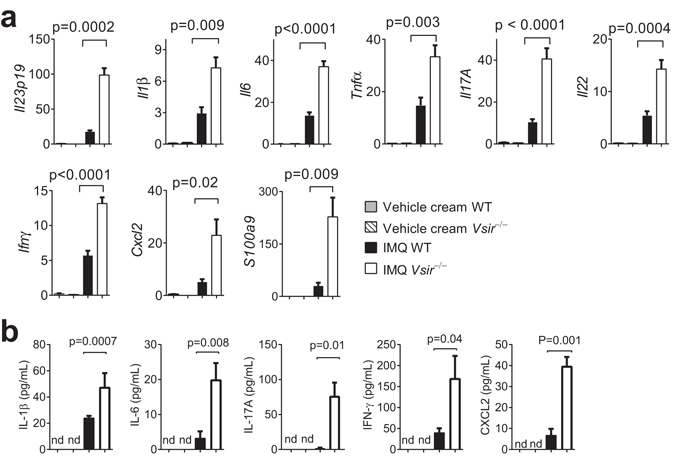
VISTA deficiency enhanced the production of inflammatory cytokines and chemokines. (**a**) WT and *Vsir*^−/−^ mice were topically treated on both ears with 3.5% IMQ cream daily for 3 days. mRNA was isolated from ear tissues. Gene expression of inflammatory cytokines and chemokines (*Il23p19*, *Il1b*, *Il6, Tnfa*, *Il17a*, *Il22*, *Ifng*, *Cxcl2*, *S100a9*) was examined by quantitative RT-PCR. The relative mRNA abundance of each gene is normalized against the control gene *Gapdh* and shown as mean ± SEM (n = 4). Representative data from three independent experiments are shown. (**b**) Serum levels of IL-1β, IL-6, IL-17A, IFN-γ, and CXCL2 in WT and *Vsir*^−/−^ mice before and after IMQ cream treatment were examined by ELISA. Data are shown as mean ± SEM (n = 4).

Exaggerated neutrophil infiltration may potentially result from an augmented production of inflammatory cytokines and chemokines^18^. To investigate the inflammatory milieu, mRNA from IMQ-treated ear skin was harvested and examined by quantitative RT-PCR (Q-PCR). A panel of psoriasis-associated genes was induced in both WT and *Vsir*^−/−^ skin following IMQ treatment (Fig. 2a). *Vsir*^−/−^ skin showed significantly higher expression of inflammatory cytokine genes *Il23p19, Il1β, Il6, Il17a, Il22, Tnfα*, and *Ifn*γ, chemokine gene *Cxcl2*, and neutrophil chemotactic gene *S100a9* (Fig. 2a). Serum protein levels of IL-1β, IL-6, IL-17A, IFN-γ, and CXCL2 were higher in *the Vsir*^−/−^ mice (Fig. 2b). Serum levels of S100A9, TNF-α, IL-22, and IL23 were very low and were not reliably detected before or after IMQ treatment (unpublished results). Serum levels of IL-22 and TNF-α have been previously reported in mice following treatment with 5% IMQ cream on the back skin^20^. To further confirm that VISTA deficiency resulted in enhanced protein production of IL-22 and TNF-α, WT and *Vsir*^−/−^ mice were treated with 3.5% IMQ cream on the shaved back skin. Serum was harvested six hours after the treatment, and the concentration of IL-22 and TNF-α in the serum was examined by ELISA. Consistently, IMQ treatment of back skin led to accumulation of higher levels of IL-22 and TNF-α in the serum of the *Vsir*^−/−^ mice (Supplementary Fig. 1). Together, these results indicate that VISTA inhibits the expression of inflammatory cytokines and chemokines in response to IMQ.

### VISTA regulates the production of IL17 by both γδ T cells and CD4^+^ Th17 cells

The IL-23/IL-17 inflammatory axis has a well-established role in the development of inflammatory and autoimmune diseases^21,22^. In psoriatic lesions, high levels of IL-17A induce the release of neutrophil chemoattractants from keratinocytes, thus amplifying the inflammatory cascade. IL-22, another key Th17 cytokine, promotes sustained epidermal acanthosis and parakeratosis^18,23,24^.

**Figure 3:**
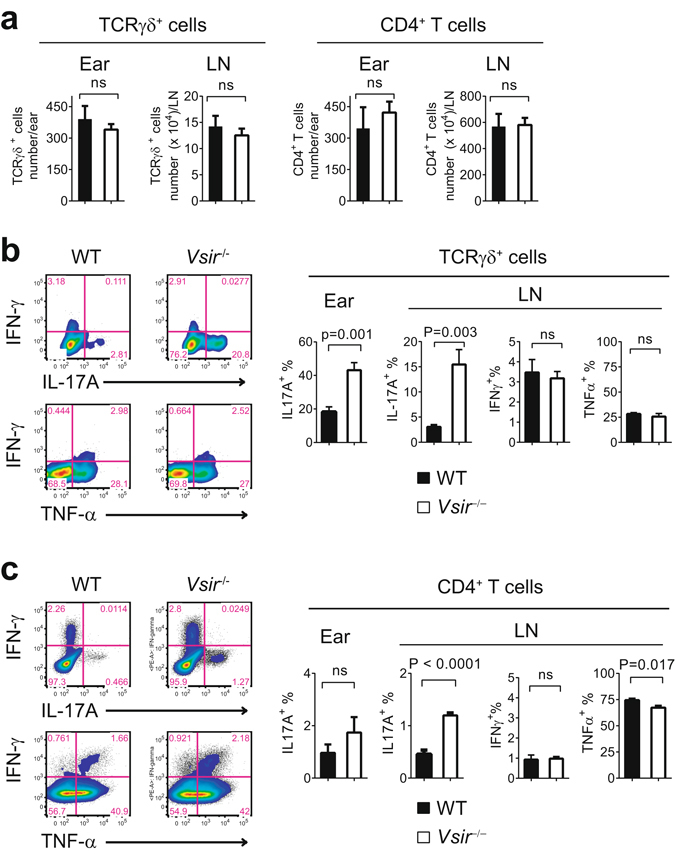
VISTA suppressed the production of IL-17A by γδ T cells and CD4^+^ Th17 cells following IMQ treatment. WT and *Vsir*^−/−^ mice were topically treated on ears with 3.5% IMQ cream daily for 3 days. Cells from ear skin and draining LN were harvested and stimulated *in vitro* with PMA and Ionomycin. The number of γδ T cells and CD4^+^ T cells, and their expression of cytokines (IL-17A, IFN-γ, and TNF-α) were examined by surface and intracellular staining, and quantified by flow cytometry. Total cell number of recovered γδ T cells and CD4^+^ T cells from ear tissue and draining LN are shown (**a**). The percentages of γδ T cells (**b**) and CD4^+^ T cells (**c**) expressing cytokines IL-17A, IFN-γ, and TNF-α were examined by flow cytometry and are shown as mean ± SEM. Representative flow plots and representative data from three independent experiments are shown.

Both γδ T cells and CD4^+^ Th17 cells are IL-17-producing effector cells that drive human psoriasis and the IMQ-induced model of psoriasiform inflammation^10,12–14^. To investigate the contribution of γδ T cells and CD4^+^ Th17 cells during the development of psoriasiform inflammation in the *Vsir*^−/−^ mice, we first examined their cell number in the ear and ear-draining LN. No significant difference was observed between WT and *Vsir*^−/−^ mice (Fig. 3a). Since *Il17a* mRNA level was significantly augmented in the *Vsir*^−/−^ skin (Fig. 2), we examined IL-17A protein expression in γδ T cells and CD4^+^ cells isolated from IMQ-treated WT and *Vsir*^−/−^ mice. *Vsir*^−/−^ γδ T cells in ear skin and draining LN produced significantly higher amount of IL-17A, but similar levels of IFN-γ and TNF-α when compared to WT cells (Fig. 3b and Supplementary Fig. 2). *Vsir*^−/−^ CD4^+^ T cells also expressed higher amount of IL-17A than WT cells in the ear and ear-draining LN, though the percentage of IL-17A positive cells was much lower than γδ T cells (Fig. 3c). Thus, both γδ T cells and Th17 cells contributed to the higher IL-17A production in IMQ-treated *Vsir*^−/−^ mice. In addition to γδ T cells and CD4^+^ T cells, CD11b^+^ myeloid cells expressed IFN-γ and TNF-α (Supplementary Fig. 3a). Higher numbers of IFN-γ and TNF-α-expressing CD11b^+^ cells were found to infiltrate IMQ-treated ears in the *Vsir*^−/−^ mice, which may contribute to the higher levels of IFN-γ and TNF-α within the inflamed ear tissues (Supplementary Fig. 3b). Similar numbers of IFN-γ and TNF-α-expressing Cd11b^+^ cells were found in the ear-draining LN (Supplementary Fig. 3c).

**Figure 4:**
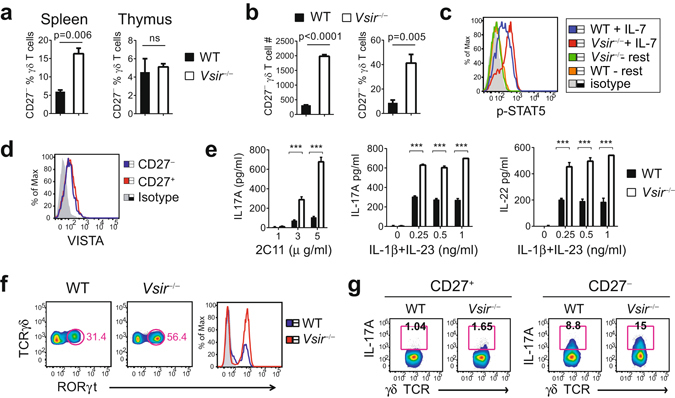
VISTA controls the peripheral homeostasis and activation of γδ T cells. (**a**) Naive γδ T cells were isolated from WT or *Vsir*^−/−^ mice and their surface expression of CD27 was examined by flow cytometry (n = 4). The percentage of the CD27^−^ subset is shown (mean ± SEM). (**b** and **c**) Naïve γδ T cells from WT and *Vsir*^−/−^ mice (n = 4) were stimulated *in vitro* with IL-7 (10 ng/ml). The number and percentage of viable CD27^−^ γδ T cells were quantified by flow cytometry after 4 days. Phosphorylated STAT5 was examined by intracellular staining and flow cytometry. (**d**) VISTA expression on CD27^+^ and CD27^−^ naïve splenic γδ T cells was examined by flow cytometry. (**e**–**g**) Naïve splenic γδ T cells were purified from WT and *Vsir*^−/−^ mice (cells pooled from 4 mice of each strain), and stimulated with either immobilized anti-CD3ε mAb (2C11) or cytokines IL-1β and IL-23. Culture supernatants were harvested after 24 hrs and the levels of IL-17A and IL-22 were examined by ELISA. Values from triplicated cultures are shown as mean ± SEM (**e**). Expression of RORγt at 24 hrs following IL-1β (0.25 ng/ml) and IL-23 (0.25 ng/ml) stimulation was examined by intracellular staining and flow cytometry (**f**). The percentage of IL-17A-producing CD27^+^ and CD27^−^ γδ T subsets was examined by flow cytometry (**g**). ***P < 0.001. Representative results from three independent experiments are shown.

To further understand the mechanisms whereby VISTA regulates the homeostasis and activation of γδ T cells, we examined the subsets of γδ T cells in naïve WT and *Vsir*^−/−^ mice^25,26^. The CD27^+^ and CD27^−^ γδ T cell subsets are developed in thymus before exiting to the periphery^25^. The CD27^−^ γδ T cells express higher levels of IL-1R and IL-23R, and are pre-committed to produce IL-17A upon TCR- or cytokine-mediated activation, whereas CD27^+^ γδ T cells predominantly produce IFN-γ^25,27^. To determine if loss of VISTA altered the development and peripheral homeostasis of γδ T cells, thymic and splenic γδ T cell subsets in WT and *Vsir*^−/−^ mice were examined. Similar percentages of total γδ T cells and the Vγ4^+^ subset were observed in the spleen, indicating an overall normal development of γδ T cells in the absence of VISTA (unpublished data). On the other hand, higher percentage of the CD27^−^ subset was present within the splenic but not thymic *Vsir*^−/−^ γδ T cells (Fig. 4a). This result indicates that VISTA regulates the peripheral homeostasis of CD27^−^ γδ T subsets.

It has been shown that IL-7 preferentially expands the IL-17A-producing CD27^−^ γδ T subset and promotes their peripheral homeostasis^28,29^. To determine whether the peripheral expansion of CD27^−^ γδ subset in *Vsir*^−/−^ mice resulted from unrestricted IL-7 receptor signaling, naive WT and *Vsir*^−/−^ γδ T cells were stimulated *in vitro* with IL-7 for 4 days, and the number of CD27^−^ γδ T cells was examined. IL-7 treatment expanded both WT and *Vsir*^−/−^ CD27^−^ γδ T subsets. However, *Vsir*^−/−^ CD27^−^ γδ T cells were hyper-proliferative than WT cells, resulting in a ~10 fold increase in viable cell number and a ~5 fold increase in percentage within the total expanded γδ T cell population (Fig. 4b). To determine how VISTA regulates the IL-7 receptor signaling, the phosphorylation status of STAT3 and STAT5 was examined, since both proteins are known to mediate IL-7 receptor signaling^30^. Our results show that higher level of phosphorylated STAT5 was induced in *Vsir*^−/−^ γδ T cells than WT cells following IL-7 stimulation (Fig. 4c). On the other hand, similar level of phosphorylated STAT3 was observed (data not shown). These results indicate that VISTA restricts the activation of STAT5 but not STAT3 downstream of IL-7R signaling in γδ T cells.

γδ T cells are activated by both TCR-specific stimuli and inflammatory cytokines such as IL-1β and IL-23^26^. Since VISTA is expressed on γδ T cells (Figs 1f and 4d), it is possible that VISTA suppresses the activation of γδ T cells in an autonomous manner. To test this, naïve splenic WT and *Vsir*^−/−^ γδ T cells were isolated and stimulated with either TCR crosslinking, or cytokines IL-1β and IL-23. *Vsir*^−/−^ γδ T cells produced more IL-17A and IL-22 than WT cells (Fig. 4e). Activated *Vsir*^−/−^ γδ T cells consistently expressed higher level of RORγt, a ROR family transcription factor that binds to and activates the *Il-17a* promoter (Fig. 4f)^31,32^. Both CD27^+^ and CD27^−^ *Vsir*^−/−^ γδ T subsets expressed more IL-17A than WT cells following IL-1β and IL-23 stimulation. These data indicate that in addition to regulating the peripheral homeostasis of CD27^−^ γδ T cells, VISTA directly controls the activation of γδ T cells (Fig. 4g).

### VISTA expression on dendritic cells suppresses IMQ-induced TLR7 signaling and IL-23 production

**Figure 5:**
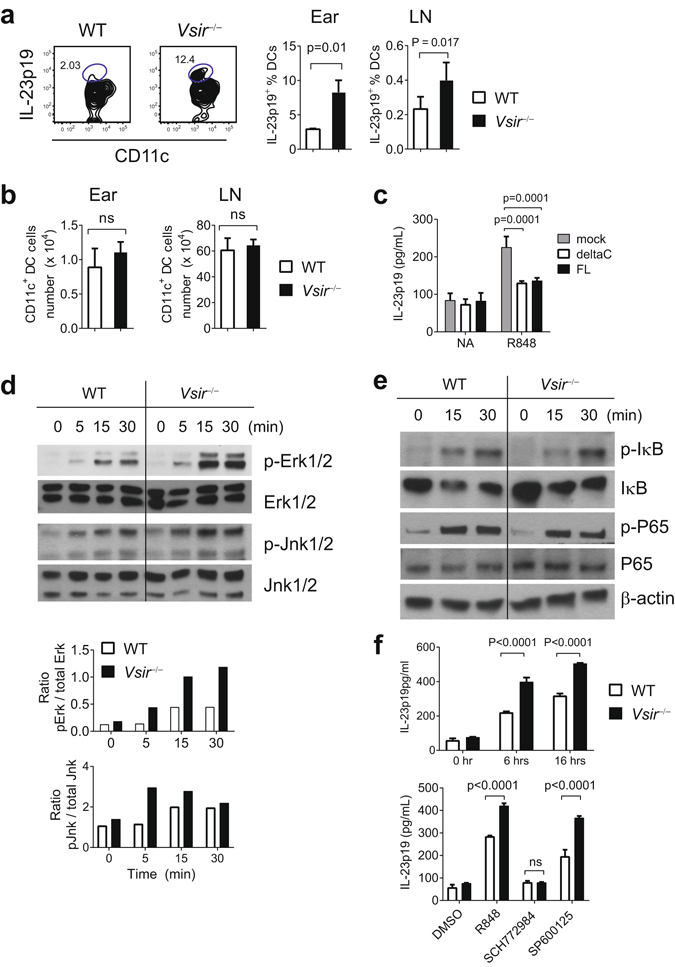
VISTA negatively regulates IMQ-induced activation of DCs and the production of IL-23. WT and *Vsir*^−/−^ mice were treated on ears with 3.5% IMQ for 3 days. Cells from ear tissues and the ear-draining cervical LNs were harvested. Cells were stimulated with PMA and Ionomycin *in vitro* for 3 hrs. The expression of IL-23p19 in CD11c^+^ DCs was examined by flow cytometry. The percentages of IL-23p19-expressing DCs were quantified and shown as mean ± SEM (n = 6) in (**a**). The number of total CD11c^+^ DCs from ear tissue and draining LN is shown as mean ± SEM (n = 5) in (**b**). To determine whether ectopic expression of VISTA suppresses TLR7-induced IL-23 production, *Vsir*^−/−^ BM-derived DC were transduced with lentivirus expressing full-length (FL), or mutant VISTA lacking the cytoplasmic tail (deltaC), or GFP control protein. After culture with GM-CSF (20 ng/ml) for 7 days, cells were stimulated with R848 (5 μg/ml) for 7 hrs. Culture supernatant was harvested and secreted IL-23p19/p40 was examined by ELISA (**c**). To examine TLR7 signaling in DCs, CD11c^+^ DCs were purified from the spleens of naïve *Rag1*^−/−^ and *Vsir*^−/−^*Rag1*^−/−^ mice, and stimulated with R848 (5 μg/ml) for indicated amount of time. Total cell lysates were prepared and examined for the levels of phosphorylated Erk1/2 and Jnk1/2 by western blotting (**d**). The ratio of phosphorylated versus total Erk1/2 and Jnk1/2 was calculated based on the total protein level from the same lysate run on a parallel gel (**d**). The activation of NF-κB signaling was examined by western blotting the level of phosphorylated and total IκB, as well as phosphorylated and total NF-κB p65 subunit (**e**). To determine whether Erk and Jnk were required for the production of IL-23, Splenic DCs were isolated from naïve WT and *Vsir*^−/−^ mice, and stimulated with R848 (5 μg/ml) in the presence of Erk1/2 inhibitor (SCH772984, 10 μM), or Jnk1/2 inhibitor (SP600125, 10 μM), or DMSO solvent control for overnight. Culture supernatant was collected and secreted IL-23p19/p40 was quantified by ELISA. Values from triplicated cultures are shown as mean ± SEM in (**f**). Representative results from two to three independent experiments were shown.

In both human psoriasis and murine models of psoriasiform inflammation, IL-23 is predominantly produced by myeloid DCs and promotes the expansion of pathogenic IL-17A-producing γδ T cells and CD4^+^ Th17 cells^17,20,24^. Since an elevated expression of *IL23* gene was observed in IMQ-treated ear skin from *Vsir*^−/−^ mice (Fig. 2), we hypothesize that VISTA expression on DCs suppresses IMQ/TLR7-induced IL-23 production. To test this hypothesis, WT and *Vsir*^−/−^ mice were treated with 3.5% IMQ on the ears for 4 days. Ear tissues were harvested and the expression of IL-23p19 in ear CD11c^+^ DCs were examined by flow cytometry. Significantly higher percentages of *Vsir*^−/−^ DCs expressed IL-23p19 protein than WT cells in ears and ear-draining LNs (Fig. 5a), whereas the number of DCs present in ears and the draining LNs was similar (Fig. 5b).

Although there was similar expression of DC activation markers such as CD80, CD86, and CD40 on naive WT and *Vsir*^−/−^ splenic DCs (Supplementary Fig. 4), it could not be formally excluded that an altered DC development in *Vsir*^−/−^ mice may contribute to the hyper-response of DCs. To directly demonstrate the role of VISTA in suppressing DC cytokine production, we ectopically expressed either full-length VISTA, or a mutant VISTA lacking the cytoplasmic tail (deltaC), or GFP control protein in GM-CSF cultured *Vsir*^−/−^ BM-derived DCs. Following stimulation with a TLR7/8 agonist R848, secreted IL-23 was examined by ELISA. Expression of both FL-VISTA and deltaC-VISTA significantly suppressed IL-23 production in *Vsir*^−/−^ BMDCs (Fig. 5c). This result strongly supports the role of VISTA in inhibiting TLR7-mediated DCs activation and IL-23 expression. Furthermore, since the cytoplasmic tail is not required for the suppressive activity of VISTA, this result indicates that VISTA engages an unknown receptor, which in turn delivers an inhibitory signal.

The *Il-23* promoter contains binding sites for AP-1 and NF-κB^33^. It has been shown that TLR4 stimulation in macrophages and DCs activates MAP kinases (Erk1/2, Jnk1/2, and p38), which are critical for the activation of transcription factor AP1 and the expression of *Il-23p19* gene^33,34^. Furthermore, Erk1/2 inhibitor suppressed IL-23 production in DCs stimulated with TLR agonists^34^. To determine if VISTA regulates the activation of NF-κB and MAPK pathways, total cell lysates were prepared from WT and *Vsir*^−/−^ splenic DCs that have been stimulated with R848 and examined by western blotting (Fig. 5d). R848 stimulation induced significantly higher levels of Erk1/2 phosphorylation and a moderately increased phosphorylation of Jnk1/2 in *Vsir*^−/−^ DCs (Fig. 5d). On the contrary, similar levels of Iκ-B degradation and phosphorylation of NF-κB p65 were observed, indicating that the NF-κB pathway was not significantly altered in the absence of VISTA (Fig. 5e). Similar levels of phosphorylated p38 were present in lysates from WT and *Vsir*^−/−^ BMDCs (unpublished data).

To further confirm the critical role of Erk1/2 in IL-23 production in DCs, WT and *Vsir*^−/−^ DCs were purified from naïve *Rag1*^−/−^ and *Vsir*^−/−^*Rag1*^−/−^ mice and stimulated *ex vivo* with R848 in the presence of inhibitors of Erk1/2 or Jnk1/2, or solvent control (Fig. 5). Consistent with our hypothesis, *Vsir*^−/−^ DCs produced higher level of IL-23p19/p40 than WT cells (Fig. 5f). Erk1/2 inhibitor completely abolished the ability of both WT and *Vsir*^−/−^ DCs to produce IL-23, whereas Jnk1/2 inhibitor was moderately effective (Fig. 5f). Taken together, these results suggest that VISTA negatively regulates TLR7 signaling and inhibits the expression of IL-23 in DCs via suppressing the activation of Erk1/2.

## Discussion

The IL-23/IL-17-mediated inflammatory axis plays a critical role in many inflammatory disorders and autoimmune diseases such as psoriasis, rheumatoid arthritis, multiple sclerosis, and inflammatory bowel disease^22^. In the current study we have demonstrated a novel role of VISTA in regulating this inflammatory axis. In the IMQ-induced psoriasis model, VISTA deficiency augmented the inflammatory responses of DCs, γδ T cells, and Th17 cells, resulting in exacerbated psoriasiform dermatitis.

In both human psoriasis and murine model of psoriasiform inflammation, one of the main initial responders are IL-23-producing DCs^17^. IL-23 promotes the expansion and activation of IL-17-producing CD4^+^ Th17 cells and γδ T cells. This inflammatory milieu recruits and activates additional effector cells such as inflammatory monocytes and neutrophils, which amplify inflammation and drive epidermal hyperplasia. Our results indicate that VISTA controls the production of IL-23 in DCs via inhibiting the activation of Erk1/2. We predict that strategies that enhance VISTA-regulated inhibitory signaling will dampen IL-23-mediated inflammatory axis and benefit the treatment of not only human psoriasis, but also other inflammatory diseases driven by IL-23.

In addition to regulating the activation of DCs, VISTA negatively regulates IL-7-mediated homeostasis of CD27^−^ γδ T cells, as well as the activation of γδ T cells in response to TCR-mediated or IL-23/IL-1β-mediated stimuli. These effects collectively contribute to the exaggerated psoriasiform inflammation in the *Vsir*^−/−^ mice. It is noted that VISTA expression on γδ T cells was upregulated within the psoriatic skin when compared to the draining LN, indicating a potential feedback mechanism whereby inflammatory cytokines or other mediators may upregulate VISTA expression to dampen inflammation.

In addition to VISTA, other immune-checkpoint proteins including Programmed death-1 (PD-1) and B and T lymphocyte attenuator (BTLA) also regulate IL17 expression in γδ T cells^35,36^. Both receptors are expressed on γδ T cells and restrict their activation. PD-1 and BTLA knockout mice developed more severe psoriasiform dermatitis in the IMQ model^35,36^. These results warrant future efforts to determine whether these immune-checkpoint proteins act synergistically to regulate the function of γδ T cells.

In addition to psoriasis, the IL-23/IL-17 inflammatory axis regulates disease development in murine experimental autoimmune encephalomyelitis (EAE) and human autoimmune disease multiple sclerosis^26,37,38^. Previous studies have shown that VISTA genetic deletion or VISTA-blocking mAb treatment exacerbated disease in the EAE model^1,7,9^. Since both IL-17-producing γδ T cells and Th17 cells have been implicated as effector cells during EAE^39,40^, our current study provides additional mechanisms whereby VISTA regulates this disease.

In the context of cancer therapy, the IL-23/IL-17 inflammatory axis regulates the inflammatory tumor microenvironment (TME). Earlier studies have demonstrated the tumor-promoting role of IL-23^41,42^, whereas both tumor-promoting and tumor-inhibitory roles of IL-17 have been reported^43–47^. Results from this study indicate that blocking VISTA promotes the inflammatory responses mediated by IL-23/IL-17, particularly in the context of TLR stimulation. Our previous study has shown that VISTA-blocking mAb synergized with a tumor peptide vaccine and TLR agonists as adjuvants^8^. Future studies are warranted to determine whether the exacerbated IL23/IL17 inflammatory axis positively or negatively contributes to the anti-tumor immunity following VISTA blockade.

In conclusion, this study reveals a novel anti-inflammatory role of VISTA through regulating the IL-23/IL-17 inflammatory axis. Our findings distinguish VISTA from other immune-checkpoint proteins CTLA-4 and PD-1, and establish VISTA as a regulator of both innate and adaptive immunity^1,4,6–9^. Therapeutic agents have been developed to harness the immune-suppressive functions of immunecheckpoint proteins. For example, a fusion protein CTLA4-Ig (Abatacept) has been used in the clinic for treating autoimmune diseases such as rheumatoid arthritis^48^. Similarly, local overexpression of PD-L1-Ig or administration of purified PD-L1-Ig has been shown to promote allograft survival in murine models^49–52^. Our study indicates that enhancing the anti-inflammatory function of VISTA may benefit the treatment of a variety of inflammatory and autoimmune disorders.

## Materials and Methods

### Mice

C57BL/6 mice were purchased from Charles River Laboratories. *Vsir*^−/−^ mice on a fully backcrossed C57BL/6 background were as described^7,9^. All animals were maintained in a pathogen-free facility at the Medical College of Wisconsin (Milwaukee, WI). All animal protocols were approved by the Institutional Animal Care and Use Committee of the Medical College of Wisconsin. All methods were performed in accordance with the relevant guidelines and regulations.

### Abs, cell lines, and reagent

Antibodies specific for γδ-TCR (GL3), CD27 (LG.3A10), IL-17A (eBio17B7), CD4 (GK1.5), CD8 (53-6.7), CD11b (M1/70), CD11c (N418), IFN-γ (XMG1.2), TNF-α (MP6-XT22), anti-CD3e (2C11) were purchased from BioLegend (San Diego, CA). Recombinant murine IL-1β and IL-23 were from Peprotech (Rocky Hill, NJ). Antibodies specific for p-STAT3, p-STAT5, p-Erk1/2 (Thr202/Tyr204), Erk1/2, p-Jnk1/2 (Thr183/Tyr185), Jnk1/2, and IκB were purchased from Cell Signaling Technology (Boston, MA). The VISTA-specific mAb was as described previously^8^. Erk1/2 inhibitor (SCH772984) was obtained from Selleckchem (Houston, TX) and Jnk1/2 inhibitor (SP600125)was obtained from Invivogen (San Diego, CA).

### Imiquimod (IMQ)-induced psoriasiform inflammation model

WT and *Vsir*^−/−^ mice were treated daily on both ears with 50 mg of 3.5% IMQ cream, which was prepared by diluting the 5% IMQ cream (Taro Pharmaceuticals, New York, NY) using the vehicle cream (Vanicream; Pharmaceutical Specialties, Cleveland, GA). Ear thickness was measured by using an Ozaki caliper (model G-A1-0.4 N) (Neill-Lavielle Supply, Louisville, KY). For histopathological analysis, H&E staining was performed on formaldehyde fixed, paraffin-embedded skin samples. Images were acquired using an INFINITY3-1C digital camera (Lumenera, Ottawa, Canada) attached to a Carl Zeiss microscope.

To quantify the amount of Munro’s abscess, the entire cross section of the ear tissue was examined. Munro’s abscess was identified as areas within the epidermis that were occupied with aggregated neutrophils. The areas of Munro’s abscess were quantified by manually defining the boundaries and measuring the area using the Image J software.

The thickness of the epidermis was also quantified using the Image J software. More than 20 random fields were measured throughout the entire cross section of the ear tissue. The increase of epidermal thickness was calculated by subtracting the average epidermal thickness of naïve ear tissues.

### Quantitative real-time PCR (RT-PCR)

Total RNA of ear skin was prepared using an RNeasy Fibrous Tissue Kit (Qiagen, Hilden, Germany). Quantitative RT-PCR was performed via StepOnePlus Real-Time PCR System (Applied Biosystems, Foster City, CA).

Primer sequences are described in the following: *Il23-p19* (forward: CCAGCAGCTCTCTCGGAATC; reverse: TCATATGTCCCGCTGGTGC)*; Il1b* (forward: CGCAGCAGCACATCAACAAGAGC; reverse: TGTCCTCATCCTGGAAGGTCCACG)*; Il6* (forward: GCAGAAAAAGGCAAAGAATC; reverse: CTACATTTGCCGAAGAGC); *Tnfa* (forward: AGGCAGTCAGATCATCTTC; reverse: TTATCTCTCAGCTCCACG); *Il17A* (forward: GAGCTTCCCAGATCACAGAG; reverse: AGACTACCTCAACCGTTCCA)*; Il22* (forward: CTG CTT CTC ATT GCC CTG TG; reverse: AGC ATA AAG GTG CGG TTG AC); *Ifn*γ (forward: GTTACTGCCACGGCACAGTCATTG; reverse: ACCATCCTTTTGCCAGTTCCTCCAG); *Cxcl2* (forward: GAAGTCATAGCCACTCTCAAGG; reverse: CTTCCGTTGAGGGACAGC); *S100a9* (forward:ATACTCTAGGAAGGAAGGACACC; reverse: TCCATGATGTCATTTATGAGGGC)*; gapdh* (forward: GTGGAGTCATACTGGAACATGTAG; reverse: AATGGTGAAGGTCGGTGTG).

### Generation of BM-derived DC and lentiviral transduction

Bone marrow (BM) cells were harvested from the femur and tibia from naive *Vsir*^−/−^ mice, and cultured in GM-CSF (20 ng/ml). On day 3, cells were infected with lentivirus expressing full-length (FL), or mutant VISTA lacking the cytoplasmic tail (deltaC), or GFP control protein. Infected cells were selected in puromycin (5 μg/mL) for additional 4 days. On day 7, cells were stimulated with R848 (5 μg/ml) for 7 hrs. Culture supernatant was harvested and secreted IL-23p19/p40 was examined by ELISA (Biolegend Inc, San Diego, CA).

### Flow Cytometry and data analysis

CD11c^+^ DCs and γδ T cells were purified from spleens of naïve WT and *Vsir*^−/−^ mice using MACS Microbead kits (Miltenyi Biotech, San Diego, CA). DCs were positively selected using the Cd11c Microbeads (130-108-338). γδ T cells were purified using the TCRγδ^+^ T Cell Isolation Kit (130-092-125). Purity was examined by flow cytometry and was typically >90%.

Cells from ear skin were harvested following digestion at 37 °C for 45 min with Liberase TL (Roche, Pleasanton, CA) and Dnase (Sigma, St Louis) to obtain single cell suspensions. To detect intracellular cytokine expression, cells were stimulated for 4 hrs in complete RPMI medium containing PMA(50 μg/ml), ionomycin (1 μg/ml), 10% FBS, 2 mM L-glutamine, 50 μM 2-mercaptoethanol, 1% penicillin-streptavidin, 1x monensin, 1x Brefeldin A (BioLegend, San Diego, CA). Cells were then fixed with 1% paraformaldehyde, permeabilized with 0.5% saponin, stained for intracellular cytokines, and analyzed by flow cytometry.

Flow cytometry was performed using an Acuri C6 or LSR II (BD Biosciences, San Jose, CA). Data were analyzed with FlowJo version 10.0.7 analysis software (Tree Star, San Carlos, CA).

### Graphs and Statistical analysis

All graphs and statistical analysis were generated using Prism 6 (GraphPad Software, Inc., San Diego, CA). Student’s t test (two tailed) or ANOVA was used for data analyses. A P-value less than 0.05 is considered as statistically significant.

## Electronic supplementary material

Dataset 1
