# Author Correction: Immune-checkpoint protein VISTA critically regulates the IL-23/IL-17 inflammatory axis

**DOI:** 10.1038/s41598-018-22151-w

**Published:** 2018-03-01

**Authors:** Na Li, Wenwen Xu, Ying Yuan, Natarajan Ayithan, Yasutomo Imai, Xuesong Wu, Halli Miller, Michael Olson, Yunfeng Feng, Yina H. Huang, Mary Jo Turk, Samuel T. Hwang, Subramaniam Malarkannan, Li Wang

**Affiliations:** 1 Department of Microbiology and Immunology, Milwaukee, WI 53226 USA; 2 Department of Dermatology, Medical College of Wisconsin, Milwaukee, WI 53226 USA; 3 Department of Medicine, Medical College of Wisconsin, Milwaukee, WI 53226 USA; 4 Department of Pediatrics, Medical College of Wisconsin, Milwaukee, WI 53226 USA; 5 Department of Blood Research Institute, Milwaukee, WI 53226 USA; 6 Department of Microbiology and Immunology, Geisel School of Medicine at Dartmouth, Hanover, New Hampshire USA; 7 Present Address: Shanghai University of Traditional Chinese Medicine, College of Pharmacy, Shanghai, 201203 P. R. China; 8 Present Address: Institute of Human Virology, University of Maryland School of Medicine, Baltimore, MD 21201 USA; 9 Present Address: Department of Dermatology, Hyogo College of Medicine 1-1, Mukogawa-cho, Nishinomiya, Hyogo 663-8501 Japan; 10 Present Address: Department of Dermatology, University of California Davis, Sacramento, CA 95816 USA; 11 Present Address: Department of Histology and Embryology, Harbin Medical University, Harbin, 150086 P. R. China

Correction to: *Scientific Reports* 10.1038/s41598-017-01411-1, published online 03 May 2017

This Article contains an error in the Acknowledgements section.

“This study was supported by research funding from NCI R01 CA164225 (L.W.), Advancing A Healthier Wisconsin Research and Education Program (AHW REP) fund (L.W.), Ann’s Hope Foundation from the Medical College of Wisconsin Cancer Center (L.W.), the Office of the Assistant Secretary of Defense for Health Affairs through the Peer Reviewed Cancer Research Program under Award No. W81XWH-14-1-0587 (L.W.), Worldwide Cancer Research Foundation (UK) research grant 16-1161 (L.W.).”

should read:

“This study was supported by research funding from NCI R01 CA164225 (L.W.), Advancing A Healthier Wisconsin Research and Education Program (AHW REP) fund (L.W.), Ann’s Hope Foundation from the Medical College of Wisconsin Cancer Center (L.W.), the Office of the Assistant Secretary of Defense for Health Affairs through the Peer Reviewed Cancer Research Program under Award No. W81XWH-14-1-0587 (L.W.), Worldwide Cancer Research (UK) research grant 16-1161 (L.W.).”

