# Supplementary material for: Immune-checkpoint protein VISTA critically regulates the IL-23/IL-17 inflammatory axis
Source: Sci Rep. 2017 May 3;7:1485. doi: 10.1038/s41598-017-01411-1 (PMC5431161; doi:10.1038/s41598-017-01411-1)

SREP-16-35562A

**Immune-checkpoint protein VISTA regulates the IL-23/IL-17 inflammatory axis**

Na Li^1*^, Wenwen Xu^1*^, Ying Yuan^1,2*^, Natarajan Ayithan^1,7^, Yasutomo Imai^3,8^, Xuesong Wu^3,9^, Halli Miller^1^, Michael Olson^1^, Yunfeng Feng^9^, Yina H Huang^9^, Mary Jo Turk^9^, Samuel T. Hwang^3,10^, Subramaniam Malarkannan^1,4,5,6^, Li Wang^1^

^1^Departments of Microbiology and Molecular Genetics, ^3^Dermatology, ^4^Medicine, ^5^Pediatrics, Medical College of Wisconsin, ^6^Blood Research Institute, Milwaukee, WI, 53226. ^9^Department of Microbiology and Immunology, Geisel School of Medicine at Dartmouth, Hanover, New Hampshire.

^2^Current address: Shanghai University of Traditional Chinese Medicine, College of Pharmacy, Shanghai 201203, P.R.China. ^7^Current address: Department of Microbiology, University of Iowa, Iowa City, IA 52242. ^8^Current address: Department of Dermatology, Hyogo College of Medicine 1-1, Mukogawa-cho, Nishinomiya, Hyogo 663-8501 Japan. ^10^Current address: Department of Dermatology, University of California Davis, Sacramento, CA 95816

* Equal contributions

**Supplementary Figure 1. Elevated levels of IL-22 and TNF-α were detected in the serum of VISTA KO mice following IMQ cream treatment.**

WT and *Vsir*^−/−^ mice were topically treated on shaved back skin ( approximate size of 2.5 cm x 3 cm) with 3.5% IMQ cream. Serum was harvested six hours after the treatment, and the levels of IL-22 and TNF-α in the serum were examined by ELISA (n=8).

**
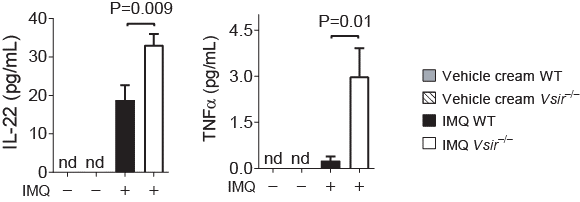
**

**Supplementary Figure 2. Histograms of cytokine expression in γδT cells and CD4^+^ T cells.** WT and *Vsir*^−/−^ mice were topically treated on ears with 3.5% IMQ cream daily for 3 days. Cells from ear skin and draining LN were harvested and stimulated *in vitro* with PMA and Ionomycin for 3 hrs. The expression of cytokines (IL-17A, IFN-γ, and TNF-α) in γδ T cells and CD4^+^ T cells, were examined by surface and intracellular staining, and quantified by flow cytometry. The histograms of cells expressing cytokines IL-17A IFN-γ, and TNF-α are shown.


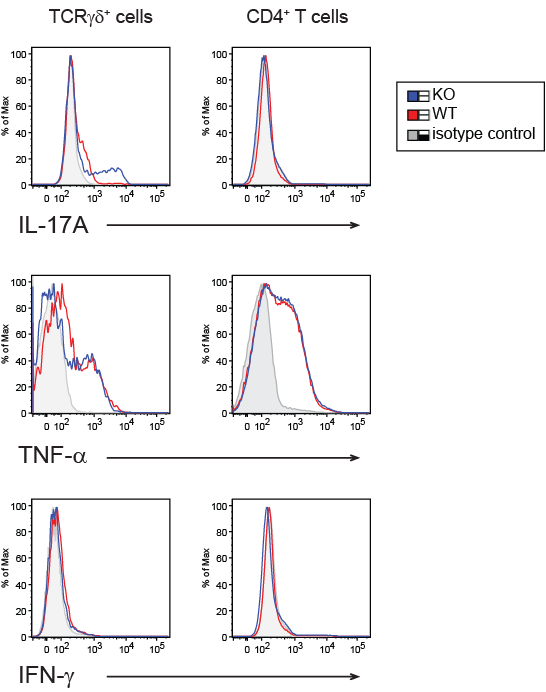


**Supplementary Figure 3. Cd11b^+^ myeloid cells express IFNγ and TNFα in IMQ-treated mice.** WT and *Vsir*^−/−^ mice were topically treated on ears with 3.5% IMQ cream daily for 3 days. Cells from ear skin and draining LN were harvested and stimulated *in vitro* with PMA and Ionomycin for 3 hrs. The expression of IFN-γ and TNF-α in CD11b^+^ myeloid cells were examined by flow cytometry. A representative flow plot of LN cells is shown in A. Cytokine-expressing CD11b^+^ cells recovered from ears and LN were enumerated and shown in B and C.


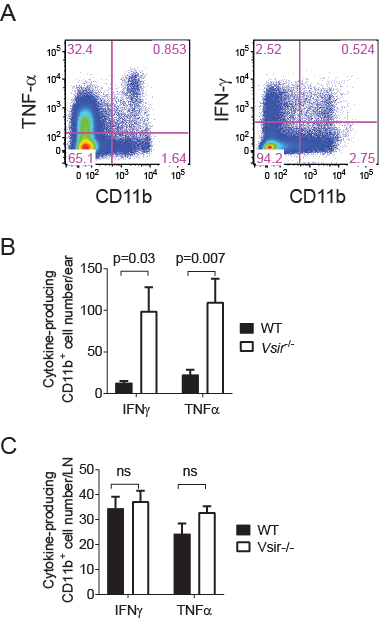


**Supplementary Figure 4. The activation status of DCs from naïve WT and *Vsir*^−/−^ mice**.

The surface expression levels of CD40, CD80, CD86 and MHCII on splenic DCs from naïve WT and *Vsir*^−/−^ mice were examined by flow cytometry.


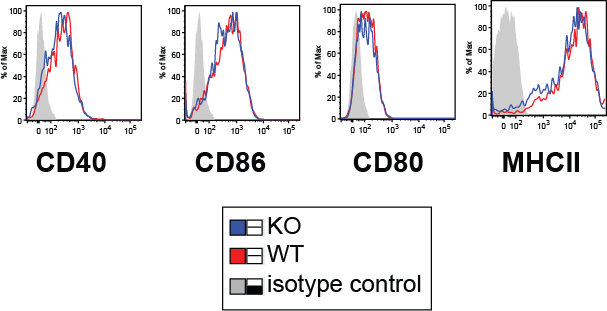

Supplement: Supplementary file 1 — Dataset 1 [file 41598_2017_1411_MOESM1_ESM.doc]
